# The bio-intelligence circuit: a hypothesis-generating systems framework linking mitochondrial stress, innate immune signaling, and autonomic regulation in chronic inflammation

**DOI:** 10.3389/fimmu.2026.1750974

**Published:** 2026-03-09

**Authors:** Parthiban Subramaniyan, Vinodhini Parthiban

**Affiliations:** 1 Clinical Translational Immunologist & Principal Investigator, Center for Inflammation and Autoimmune Research (CIAR), Under Registration Process (NVP Foundation), Thanjavur, Tamil Nadu, India; 2 M. Tech (Biotechnology), Center for Inflammation and Autoimmune Research (CIAR), Thanjavur, Tamil Nadu, India

**Keywords:** bio-intelligence circuit, chronic inflammation, hypothesis-generating framework, immune dysregulation, informational bio-recalibration, mitochondrial bioenergetics, nuclear receptor coordination, systems biology

## Abstract

Chronic inflammatory and autoimmune conditions frequently manifest as multi-organ dysfunction without a single explanatory lens that integrates metabolic stress, innate immune activation, transcriptional control, and autonomic regulation. Here, we propose the Bio-Intelligence Circuit (BIC) as a hypothesis-generating systems framework connecting mitochondrial dysfunction, LPS–TLR4–NF-κB innate immune signaling, nuclear receptor dysregulation, and vagal reflex imbalance as interacting regulatory failure patterns that may sustain chronic inflammatory states. The central hypothesis is that loss of coordinated energetic, immune-sensing, and neuro-autonomic regulation sustains a self-reinforcing dysregulation loop that amplifies inflammatory signaling, impairs regulatory restraint, and limits recovery potential. Within this framework, we introduce Informational Bio-Recalibration (IBR) as a hypothesis-generating transition sequence in which improvement of mitochondrial bioenergetics and redox buffering, attenuation of excessive TLR4 signaling, restoration of nuclear receptor transcriptional coordination, and rebalancing of autonomic tone may together shift the system toward resolution-permissive physiology. This article does not report interventional outcomes; rather, it provides a structured conceptual model and testable predictions to guide future experimental validation across inflammatory and immune-mediated phenotypes.

## Graphical Abstract

Conceptual overview of Informational Bio-Recalibration (IBR) within the Bio-Intelligence Circuit framework. The schematic illustrates a regulatory transition from mitochondrial degeneration—characterized by ΔΨm loss, elevated ROS, and NF-κB signaling—to coordinated mitochondrial–nuclear–neural integration. Restoration of PGC-1α/TFAM-mediated transcriptional regulation and engagement of vagal–α7-nAChR–IL-10 signaling are depicted as convergent processes contributing to systemic regulatory coherence and multi-organ homeostasis. 
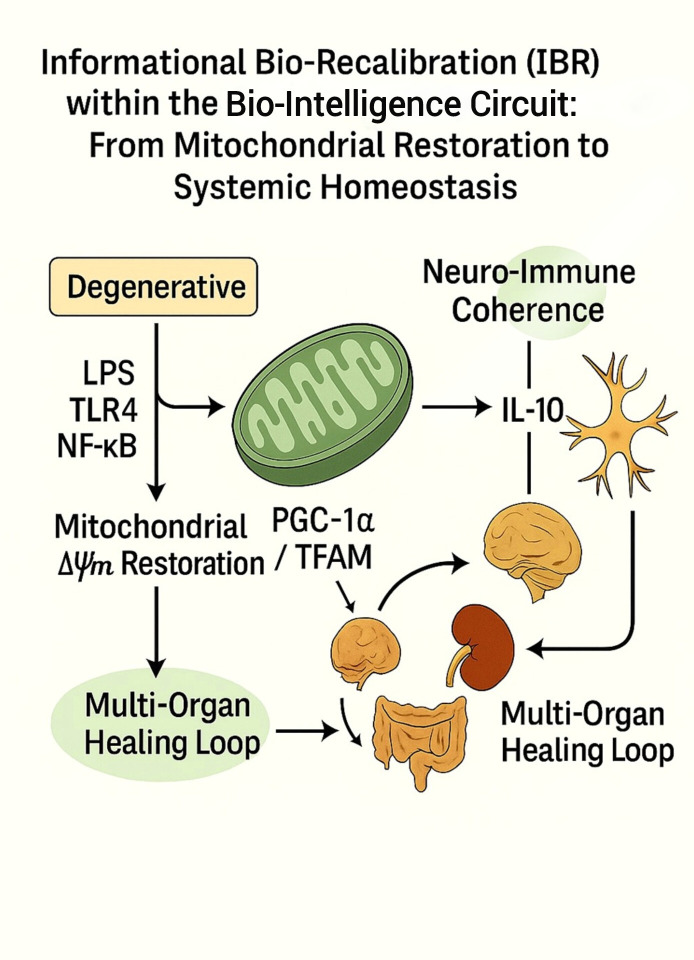

## 1 Introduction

Chronic inflammatory and autoimmune conditions commonly present as multi-organ dysfunctions that evolve over time without a single dominant pathological driver. Despite major advances in immunology, metabolism, and neurobiology, many chronic inflammatory states remain incompletely explained by isolated pathway-centric models, as patients frequently exhibit persistent immune activation, metabolic stress, autonomic imbalance, and impaired resolution mechanisms spanning multiple biological systems (1–4). This fragmentation has motivated increasing interest in integrative and systems-level perspectives capable of capturing how regulatory interactions across biological domains contribute to inflammatory persistence (5–7).

Emerging evidence indicates that mitochondrial bioenergetic stress plays a central role in shaping immune responsiveness, redox balance, and cellular resilience. Mitochondrial dysfunction has been associated with altered innate immune signaling, impaired resolution capacity, and sustained inflammatory tone, particularly through its influence on pattern recognition receptor activation and downstream transcriptional programs (8–10). In parallel, innate immune sensing pathways such as lipopolysaccharide-mediated TLR4 activation and NF-κB signaling serve as critical nodes linking metabolic stress to cytokine amplification and immune dysregulation (11–13). While these pathways are well characterized individually, their coordinated behavior within broader regulatory networks remains less clearly conceptualized in chronic disease contexts.

Nuclear receptor systems, including vitamin D receptor (VDR) and peroxisome proliferator-activated receptors (PPARs), further integrate metabolic state, immune signaling, and transcriptional restraint. Dysregulation of these transcriptional regulators has been implicated in prolonged inflammatory signaling, impaired immune tolerance, and altered cellular adaptation to stress (14–16). At the systems level, failure of nuclear receptor–mediated transcriptional coordination may amplify immune–metabolic disturbances initiated upstream, thereby reinforcing chronic inflammatory trajectories rather than facilitating resolution (17, 18).

Autonomic regulation, particularly vagal signaling, represents another critical regulatory layer influencing immune tone and inflammatory balance. Experimental and clinical literature demonstrates that vagal pathways modulate inflammatory responses through cholinergic anti-inflammatory mechanisms, neuro–immune reflexes, and central–peripheral feedback loops that influence cytokine production and immune cell activity (14–17). From an integrative perspective, persistent mitochondrial energetic stress, excessive innate immune activation, and nuclear receptor dysregulation may collectively impair autonomic coordination, resulting in vagal dysregulation that reflects failure of systemic regulatory balance rather than primary neural pathology (18–20). In this view, autonomic imbalance emerges as a system-level indicator of regulatory failure across interconnected biological domains.

Together, these observations suggest that chronic inflammation may be sustained not by a single dominant pathway but by coordinated regulatory failure patterns spanning metabolic, immune, transcriptional, and neuro-autonomic systems. However, existing models often lack a unifying conceptual structure that explains how these domains interact dynamically to reinforce inflammatory persistence or constrain recovery (5–7).

In this context, the present work introduces the Bio-Intelligence Circuit (BIC) as a systems- level framework intended to organize and interpret regulatory interactions across mitochondrial energetics, innate immune signaling, nuclear receptor transcriptional control, and autonomic regulation in chronic inflammatory states.

In this framework, bio-intelligence reflects coordinated regulatory coherence across mitochondrial energetics, immune signaling, transcriptional regulation, and autonomic integration. Informational processing refers to the continuous integration of biochemical, neural, and immune-derived signals that shape system-wide regulatory states.

Rather than proposing a prescriptive intervention or a fully constrained mechanistic model, BIC conceptualizes chronic inflammation as an emergent consequence of interacting regulatory failure patterns that impair adaptive coordination across biological scales. By integrating established biological pathways discussed above, this framework aims to clarify how disruptions in energetic capacity, immune sensing, transcriptional regulation, and autonomic feedback may interact to sustain inflammatory persistence. Accordingly, BIC is positioned to support conceptual application, hypothesis generation, and experimental prioritization, thereby guiding future systems-level and translational investigations without asserting interventional efficacy.

## 2 Conceptual definitions used in the BIC framework

### 2.1 Bio-intelligence

In this manuscript, “bio-intelligence” refers to systems-level regulatory coherence emerging from coordinated interactions among mitochondrial energetics, immune signaling, transcriptional regulation, and neuro-autonomic integration. This term is used strictly in a biological and measurable sense, reflecting adaptive regulatory capacity rather than cognitive or teleological properties.

### 2.2 Informational processing

“Informational processing” describes the continuous integration and interpretation of biochemical, neural, and immune-derived signals that shape transcriptional responses, metabolic adaptation, and immune regulation across physiological systems.

## 3 Operational interpretation and practical applicability of the BIC framework

Within this framework, the present model is intended as a mechanistic interpretive scaffold rather than a prescriptive intervention. Informational Bio-Recalibration (IBR) is conceptualized as a regulatory transition process emerging from coordinated shifts in mitochondrial, immune, and transcriptional dynamics. From a systems perspective, initiation of IBR is proposed to occur through partial resolution of upstream stress signals, including reduction of sustained immune load, improvement in mitochondrial energetic and redox balance, and restoration of transcriptional responsiveness. These shifts are not treated as isolated corrective events but as interdependent regulatory adjustments that collectively permit movement toward resolution-permissive physiology.

The relative sequencing emphasized within the BIC framework reflects regulatory dependency rather than linear causation. Although mitochondrial energetic state is emphasized in several illustrative sequences due to its integrative influence on transcriptional and immune responsiveness, the BIC is not intended to imply a single initiating node. In different physiological or pathological contexts, dysregulation may originate from persistent immune activation, autonomic imbalance, transcriptional drift, or metabolic stress, each of which can propagate constraint across the circuit. The framework therefore accommodates multiple entry points into regulatory failure, with mitochondrial dysfunction representing one common but not exclusive convergence domain. This non-linear architecture reinforces the view that chronic inflammatory states emerge from interacting disturbances rather than a fixed causal sequence.

Mitochondrial energetic state influences transcriptional capacity and immune sensing thresholds, while nuclear receptor coordination shapes transcriptional restraint and adaptive signaling. Autonomic (vagal) regulation is viewed as an emergent integrative output that reflects successful upstream coordination rather than a primary driver of recalibration. At the same time, autonomic regulation is not only an emergent output but also a feedback-active component within the circuit. Persistent autonomic imbalance can influence mitochondrial energetic efficiency, immune sensing thresholds, and transcriptional responsiveness, thereby reinforcing or constraining system-wide regulatory states. Accordingly, the relationship between autonomic tone and upstream metabolic–immune coordination is best understood as bidirectional and potentially self-reinforcing, particularly in chronic inflammatory conditions.

While vagal cholinergic signaling is emphasized as a central integrative pathway within the BIC framework, emerging evidence suggests that sympathetic–splenic pathways also contribute to systemic immune modulation. These complementary autonomic routes may operate in parallel, jointly influencing cytokine balance and inflammatory tone. Within this framework, neuro-immune regulation is conceptualized as a hybrid parasympathetic–sympathetic architecture rather than a strictly single-pathway model.

Taken together, the proposed sequence—energetic stabilization, transcriptional re-coordination, and autonomic integration—represents a conceptual ordering framework intended to guide interpretation rather than imply a fixed linear progression. Within the BIC framework, these processes are understood as recursive, state-dependent, and dynamically interactive, with bidirectional feedback across regulatory domains.

Accordingly, the figures are intended as schematic conceptual guides and should not be interpreted as strict directional pathways.

Importantly, the BIC framework distinguishes regulatory restoration from pathway suppression. IBR does not imply attenuation of protective immune function or inhibition of physiological inflammatory responses. Instead, it emphasizes re-establishment of signal discrimination, energetic sufficiency, and feedback coherence across biological systems. Within this framing, reduction of chronic inflammatory tone arises from improved regulatory alignment rather than immune suppression, preserving host defense while limiting maladaptive inflammatory persistence.

Accordingly, the model is positioned as broadly applicable but context-sensitive across inflammatory and autoimmune states, where adaptive immune mechanisms may further shape regulatory plasticity without altering the core regulatory architecture proposed.

Within the BIC framework, the ‘stimulus’ is not conceptualized as an external agent or intervention, but as a change in internal regulatory conditions that permits resolution-permissive signaling. Such stimuli may include reductions in energetic stress, restoration of transcriptional responsiveness, or normalization of immune sensing thresholds, each of which is supported by established biological evidence. The regulatory response emerges from improved system coordination rather than direct pathway activation.

## 4 Conceptual quantification and assessment proxies

- Mitochondrial energetic state → ATP availability, redox balance, ΔΨm-related indicators.
- Innate immune load → CRP trends, cytokine balance patterns.
- Nuclear receptor coordination → VDR- or PPAR-linked transcriptional responsiveness.
- Autonomic output → HRV patterns, anti-inflammatory cytokine bias (e.g., IL-10).

These indicators are presented as illustrative proxies to support pattern-based evaluation within the BIC framework and do not represent diagnostic thresholds or required measurements.

## 5 Conceptual architecture of the bio-intelligence circuit

### 5.1 Overview

The BIC is proposed as a distributed regulatory architecture that coordinates energetic capacity, immune sensing, transcriptional governance, and autonomic integration to maintain biological signal coherence. Rather than functioning as a linear cascade, the BIC operates as a closed-loop, state-dependent circuit, in which perturbations in one domain propagate constraint or distortion across others. Chronic inflammatory and multi-system disorders are conceptualized as sustained failure states of this circuit, characterized by loss of energetic sufficiency, impaired signal discrimination, and weakened regulatory restraint.

### 5.2 Core circuit components

#### 5.2.1 Mitochondrial energetic–redox module

This component represents the energetic substrate of regulation. Mitochondrial ATP availability, redox buffering capacity, and membrane potential stability define the system’s ability to support transcriptional activity, immune resolution, and adaptive signaling. Energetic insufficiency is proposed to lower regulatory thresholds, rendering immune and transcriptional systems more noise-sensitive and less discriminative.

Functional role:

- Sets energetic limits for immune resolution and transcriptional responsiveness.
- Buffers oxidative stress generated during immune activation.
- Determines recovery capacity following inflammatory demand.

#### 5.2.2 Innate immune sensing and load module

This module encompasses pattern recognition, inflammatory tone, and immune load signaling. Within the BIC framework, innate immune activation is not inherently pathological; rather, pathology arises when immune signaling persists beyond resolution capacity due to energetic or regulatory constraints.

Functional role:

- Detects internal and external perturbations.
- Generates adaptive inflammatory responses.
- Requires downstream regulatory restraint to prevent chronic activation.

#### 5.2.3 Nuclear receptor–transcriptional coordination module

Nuclear receptors function as transcriptional governors, integrating metabolic, hormonal, and immune signals into coherent gene expression programs. Effective coordination within this module enables appropriate inflammatory restraint, metabolic adaptation, and repair signaling. Dysregulation leads to transcriptional drift, impaired feedback inhibition, and prolonged inflammatory signaling.

Functional role:

- Translates energetic and immune inputs into regulated transcription.
- Maintains adaptive restraint over inflammatory gene expression.
- Coordinates metabolic–immune gene programs.

#### 5.2.4 Neuro-autonomic integration module

Autonomic regulation, particularly vagal signaling, is conceptualized as an emergent integrative output of upstream coordination rather than an isolated control node. Stable autonomic tone reflects successful energetic, immune, and transcriptional alignment, while dysautonomia signals unresolved upstream conflict within the circuit.

Functional role:

- Reflects overall circuit coherence.
- Facilitates resolution-phase immune signaling.
- Supports systemic recovery and homeostatic stabilization.

### 5.3 Interdependencies and circuit logic

#### 5.3.1 Regulatory dependency over linear causation

The BIC architecture emphasizes regulatory dependency, wherein the functional state of each component constrains or enables others. For example, mitochondrial energetic sufficiency governs transcriptional capacity and immune resolution thresholds, while transcriptional coordination determines whether immune activation remains adaptive or becomes self-perpetuating.

#### 5.3.2 Bidirectional coupling

Interactions within the BIC are bidirectional and recursive:

- Immune activation increases energetic demand.
- Energetic insufficiency impairs transcriptional restraint.
- Transcriptional dysregulation prolongs immune signaling.
- Persistent immune signaling destabilizes autonomic output.

These couplings generate self-reinforcing loops when unresolved, accounting for chronicity and multi-organ involvement.

#### 5.3.3 Emergent autonomic output

Autonomic regulation is not positioned as a primary driver but as a systems-level readout of circuit integrity. Restoration of vagal tone is therefore interpreted as evidence of upstream recalibration rather than a standalone therapeutic target.

#### 5.3.4 Failure states and chronic dysregulation

Within the BIC framework, chronic inflammatory and immune-mediated conditions represent stable maladaptive attractor states of the circuit. These states are characterized by:

- Reduced energetic margin.
- Elevated immune signaling noise.
- Impaired transcriptional restraint.
- Fragmented autonomic integration.

Importantly, these failure states persist not due to irreversible damage, but due to loss of coordinated regulation, rendering them theoretically reversible under resolution-permissive conditions.

### 5.4 Relationship to informational bio-recalibration

IBR is positioned as a state transition within the BIC, enabled when sufficient energetic capacity, transcriptional responsiveness, and immune discrimination are partially restored. IBR does not impose directionality on the circuit; rather, it reflects the circuit’s regained ability to self-correct, re-prioritize signals, and exit maladaptive regulatory states.

### 5.5 Architectural scope and intent

This architectural description is intended to guide hypothesis generation, systems-level interpretation, and future experimental design, rather than to prescribe intervention strategies or diagnostic criteria.

## 6 Hypothesis generation and testable predictions

The following hypotheses are derived from the conceptual architecture and interpretive logic of the BIC and are intended to guide future observational and experimental inquiry rather than prescribe intervention strategies.

The BIC framework is advanced as a hypothesis-generating systems model, intended to organize observations across energetic, immune, transcriptional, and autonomic domains and to support testable predictions regarding regulatory state transitions. Rather than asserting direct causality or intervention efficacy, the framework proposes conditional relationships that can be examined empirically using non-invasive, longitudinal, and pattern-based assessments.

### 6.1 General hypothesis

Chronic inflammatory and immune-mediated conditions represent stable maladaptive regulatory states of the BIC, maintained by constrained energetic capacity, impaired transcriptional restraint, and unresolved immune signaling. Partial restoration of energetic sufficiency and transcriptional responsiveness permits endogenous regulatory recalibration, resulting in reduced inflammatory persistence without suppression of protective immune function.

### 6.2 Domain-specific testable predictions

#### 6.2.1 Energetic–immune dependency

Prediction:

Individuals exhibiting reduced mitochondrial energetic margin (e.g., impaired ATP availability or redox imbalance) will demonstrate lower thresholds for sustained innate immune activation, reflected by persistent low-grade inflammatory markers.

Testable pattern:

Improvement in energetic indicators is predicted to precede or coincide with normalization of inflammatory tone, rather than follow it.

#### 6.2.2 Transcriptional restraint as a resolution gate

Prediction:

Markers of nuclear receptor coordination (e.g., VDR- or PPAR-linked transcriptional responsiveness) will correlate more strongly with resolution of chronic inflammation than absolute suppression of pro-inflammatory cytokines.

Testable pattern:

Restoration of transcriptional responsiveness will associate with:

- Reduced inflammatory persistence.
- Preserved acute immune reactivity.

This supports regulation rather than immune suppression.

#### 6.2.3 Autonomic output as an emergent readout

Prediction:

Improvement in autonomic indicators (e.g., HRV patterns, parasympathetic bias) will occur secondary to upstream energetic and transcriptional coordination, not as an isolated primary change.

Testable pattern:

Autonomic stabilization will track with:

- Reduced inflammatory noise.
- Improved recovery dynamics rather than acting as an independent driver.

#### 6.2.4 State-dependent, not linear, transitions

Prediction:

Changes across BIC domains will exhibit non-linear, threshold-dependent behavior, consistent with transition between regulatory states rather than gradual linear improvement.

Testable pattern:

Small improvements in upstream constraints may yield disproportionate downstream stabilization, consistent with attractor-state exit rather than dose-response dynamics.

### 6.3 Quantitative and methodological implications

Within the BIC framework, quantitative assessment is proposed to focus on pattern relationships and temporal sequencing, rather than fixed thresholds or single biomarkers. Relevant approaches may include:

- Longitudinal trend analysis rather than cross-sectional values.
- Multivariate correlation across energetic, immune, transcriptional, and autonomic indicators.
- Non-invasive monitoring strategies suitable for repeated measurement Importantly, these assessments are intended to evaluate regulatory coherence, not to define diagnostic cutoffs or therapeutic targets.

### 6.4 Falsifiability and boundary conditions

The BIC framework is falsifiable under the following conditions:

- If sustained inflammatory resolution occurs without corresponding changes in energetic or transcriptional indicators
- If immune suppression alone consistently restores long-term regulatory stability.
- If autonomic modulation reliably precedes upstream coordination across diverse contexts.

Failure to observe the predicted dependencies would argue against the proposed circuit logic.

### 6.5 Scope and intent

These hypotheses are presented to guide future experimental design, observational studies, and systems-level analyses. The BIC framework does not prescribe interventions or clinical protocols but instead provides an integrative scaffold for interpreting how multi-domain regulatory alignment—or misalignment—shapes chronic inflammatory persistence and resolution.

## 7 Conceptual application pathways and translational scope of the bio-intelligence circuit

### 7.1 Conceptual role of the bio-intelligence circuit

The BIC is intended to function as a decision-organizing and interpretive framework for understanding chronic inflammatory dysregulation across biological systems, rather than as a prescriptive interventional model. Its primary role is to assist in identifying dominant regulatory failure domains and to clarify how disturbances across metabolic, immune, transcriptional, and autonomic layers may interact to sustain inflammatory persistence. In this context, BIC provides a structured lens through which complex, multi-system inflammatory phenotypes can be interpreted, enabling hypothesis generation and prioritization of experimental or translational focus.

The translational relevance of this framework will depend on systematic empirical validation across defined biological contexts. Future investigations should delineate its predictive scope, boundary conditions, and applicability across heterogenous inflammatory phenotypes. Such evaluation will clarify the operational limits and translational potential of coordinated mitochondrial, immune, and neural regulation.

### 7.2 Interpretive workflow within the BIC framework

Within the BIC framework, chronic inflammation is conceptualized as an emergent outcome of coordinated regulatory imbalance, rather than isolated pathway dysfunction. Conceptual application of BIC involves evaluating the relative contribution and interaction of key regulatory domains, including mitochondrial energetic state, innate immune signaling tone, nuclear receptor transcriptional coordination, and autonomic (vagal) regulation. Rather than prescribing a fixed sequence of correction, BIC supports flexible interpretation of which regulatory domain appears dominant in a given context, acknowledging that chronic inflammatory states may arise through heterogeneous but convergent failure patterns.

This interpretive workflow emphasizes pattern recognition over pathway isolation, allowing investigators to examine how upstream energetic stress, immune sensing overload, transcriptional dysregulation, or autonomic imbalance may collectively constrain resolution capacity.

Importantly, these modes of modulation refer to domains of regulatory influence rather than specific interventions, ensuring compatibility with physiological safety and ethical translational boundaries.

### 7.3 Adaptive modulation versus suppressive intervention

A core principle underlying the BIC framework is the distinction between adaptive regulatory modulation and pathway-specific suppression. Rather than aiming to inhibit immune activity or dampen inflammatory signaling indiscriminately, BIC emphasizes restoration of physiological coordination across regulatory systems. Within this framing, inflammatory persistence is viewed as a consequence of impaired signal discrimination and regulatory restraint, rather than excessive immune function per se.

Accordingly, BIC does not imply suppression of protective immune responses but instead frames resolution as emerging from improved alignment between energetic capacity, immune sensing, transcriptional control, and autonomic feedback. This perspective supports preservation of physiological immune defense while reducing chronic inflammatory “noise” that characterizes dysregulated states.

### 7.4 Modes of modulation: non-prescriptive classification

The BIC framework does not mandate specific interventional modalities, nor does it distinguish rigidly between invasive and non-invasive approaches. Instead, it provides a non-prescriptive classification of modulation domains, reflecting where regulatory influence may occur rather than how it must be delivered. These domains may include behavioral and lifestyle factors, metabolic and nutritional influences, neuro-autonomic regulation, and adjunctive pharmacological modulation. By avoiding prescriptive pathways, BIC remains adaptable across experimental, clinical, and population-level contexts, while maintaining its role as a conceptual scaffold for understanding regulatory dynamics rather than a treatment algorithm.

### 7.5 Conceptual quantification and evaluation logic

Evaluation within the BIC framework is inherently pattern-based, relying on convergence across multiple biological indicators rather than single quantitative thresholds. Regulatory domains implicated within BIC may be assessed using established proxy measures reflective of energetic state, immune signaling balance, transcriptional responsiveness, and autonomic tone. Importantly, no single biomarker is sufficient to define system-level regulation; instead, coordinated trends across domains provide insight into regulatory coherence or failure.

This approach supports future experimental validation by guiding selection of multimodal biomarkers appropriate to the regulatory domain under investigation, without asserting quantitative criteria at the current conceptual stage.

### 7.6 Scope and boundary conditions

The Bio-Intelligence Circuit is proposed as a conceptual and hypothesis-generating framework and does not constitute a validated clinical tool or interventional protocol. It is not intended to explain acute inflammatory responses to infection, trauma, or toxic injury, nor to replace established therapeutic strategies. Rather, BIC is designed to guide interpretation of chronic inflammatory dysregulation and to inform future experimental and translational research aimed at understanding system-level regulatory failure and recovery dynamics.

## 8 Methodology and conceptual framework

### 8.1 Data integration and observational basis

The proposed IBR framework synthesizes multidisciplinary literature—including mitochondrial bioenergetics, innate immunity, and neurophysiology—with longitudinal, de-identified observational patterns derived from translational clinical environments. This synthesis is conceptual rather than interventional. Biological signal interpretation was informed by trends in commonly used biochemical markers (CRP, ESR, IgE, Vitamin D, lipid profiles) as indicators of systemic inflammatory tone, without application of diagnostic thresholds or treatment decisions.

### 8.2 Conceptual mapping and interpretive logic

Rather than defining a procedural workflow, the following stages describe conceptual states used to organize mechanistic interpretation within the BIC framework:

#### 8.2.1 Degenerative state recognition

Characterized by elevated inflammatory markers and clinical fatigue patterns, reflecting loss of regulatory coordination.

#### 8.2.2 Energetic constraint mapping

Conceptual association of inflammatory persistence with mitochondrial stress, redox imbalance, and Δψm instability.

#### 8.2.3 Regulatory coupling interpretation

Integration of TLR4–MyD88–NF-κB signaling with nuclear receptor and vagal feedback loops to explain sustained inflammatory signaling.

#### 8.2.4 Informational bio-recalibration framing

Identification of a transitional regulatory phase in which energetic, transcriptional, and neuro-immune coordination begin to re-align, enabling movement toward resolution-permissive physiology.

### 8.3 Ethical and translational alignment

All interpretations presented are conceptual and intended for hypothesis generation. No clinical interventions or experimental protocols are reported. The framework is positioned as a pre-validation systems model designed to inform future studies conducted under formal institutional ethics approvals and regulatory standards.

## 9 Mechanistic overview: from lps-driven degeneration to bio-intelligent regeneration

The following mechanistic phases are presented to explain regulatory state transitions within the Bio-Intelligence Circuit and are not intended to represent temporal intervention stages.

### 9.1 Degenerative phase: LPS–TLR4–MyD88 signaling cascade

In chronic inflammatory conditions, lipopolysaccharides (LPS) activate toll-like receptor 4 (TLR4) on immune and epithelial cells, initiating the MyD88–NF-κB cascade. This promotes transcription of pro-inflammatory cytokines such as TNF-α, IL-1β, and IL-6, leading to reactive oxygen species (ROS) accumulation and impaired mitochondrial Δψm (16–18). The mitochondrial outer-membrane permeability transition further amplifies cytokine release, creating a feed-forward inflammatory loop that sustains oxidative stress (19, 20).

While LPS–TLR4–NF-κB signaling is presented here as a representative and well-characterized model of innate immune activation, the Bio-Intelligence Circuit framework does not assume this pathway to be a universal driver across all chronic inflammatory conditions. Instead, it serves as an illustrative example of how persistent innate immune sensing can interact with mitochondrial energetics, transcriptional coordination, and autonomic regulation within a broader systems-level architecture.

### 9.2 Transitional phase: activation of PGC-1α and TFAM

**Figure 1:**
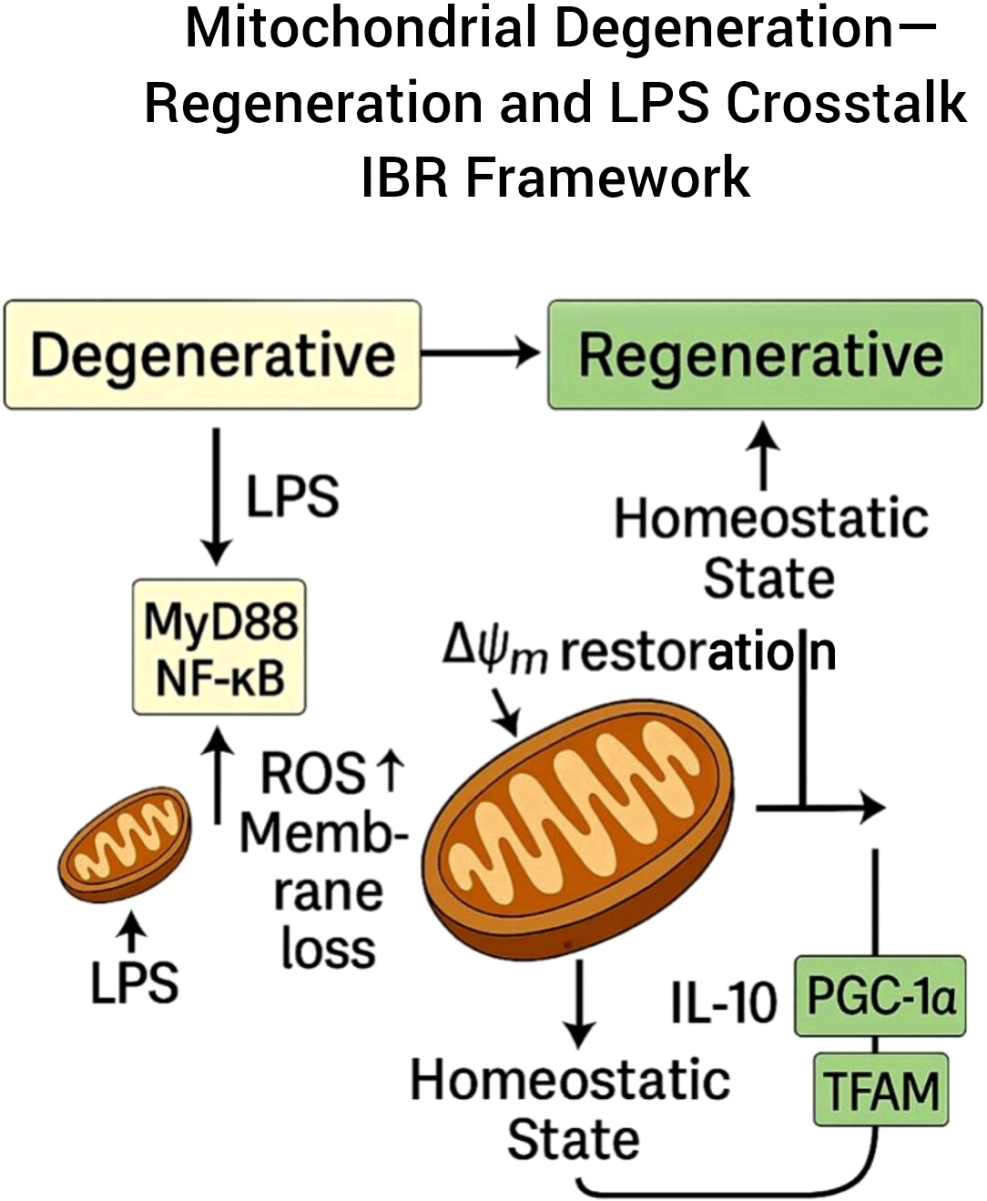
Mitochondrial degeneration–regeneration transition within the IBR framework.

During the intermediate phase, cellular stress signaling shifts toward repair mechanisms. PGC-1α (peroxisome proliferator-activated receptor-γ coactivator 1-α) and TFAM (mitochondrial transcription factor A) act as master regulators of mitochondrial biogenesis. Their up-regulation restores mitochondrial copy number and electron-transport-chain efficiency while decreasing ROS (21–23). This phase corresponds to a proposed initiation point of Informational Bio-Recalibration (IBR), characterized by restoration of coordinated energy-informational flow. This transition from mitochondrial degeneration to redox restoration is illustrated in **Figure 1**.

Schematic representation of the proposed transition from an inflammatory degenerative state to a regenerative homeostatic state. In the degenerative phase (left), lipopolysaccharide (LPS) signaling activates the MyD88–NF-κB pathway, promoting reactive oxygen species (ROS) accumulation and mitochondrial membrane destabilization. This is associated with impaired mitochondrial function. Restoration of mitochondrial membrane potential (ΔΨm) marks the initiation of a regenerative transition. In the regenerative phase (right), IL-10 signaling and activation of mitochondrial biogenesis regulators (PGC-1α and TFAM) contribute to re-establishment of redox balance and homeostatic stability. Arrows indicate conceptual directional transitions within the proposed model.

### 9.3 Regenerative phase: neuro-immune and nuclear convergence

**Figure 2:**
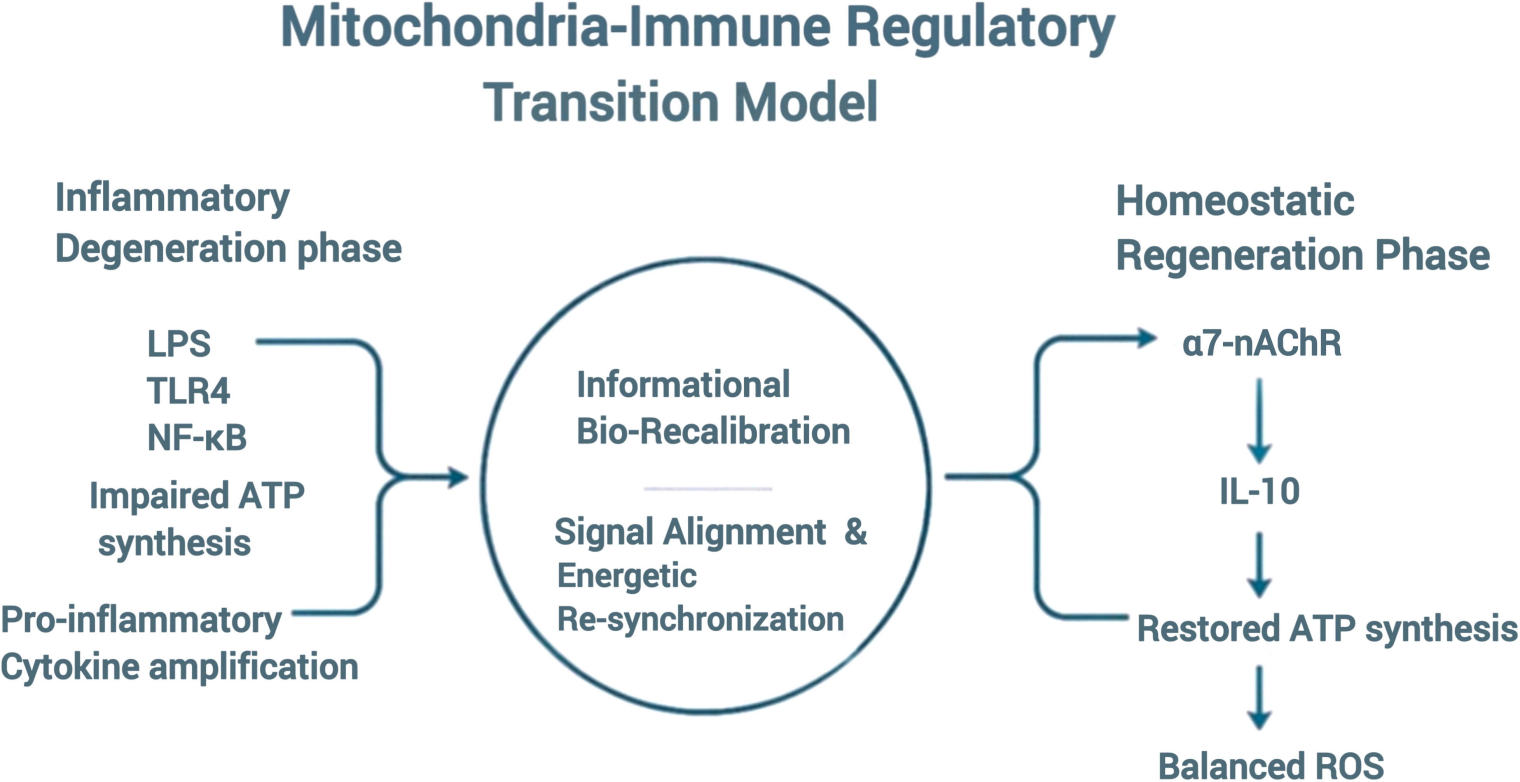
Mitochondria-Immune Axis during Informational Bio-Recalibration (IBR).

As nuclear receptors (VDR, PPAR-γ) regain transcriptional coherence, downstream anti-oxidative and metabolic genes are expressed. Concurrently, the vagal anti-inflammatory reflex triggers α7-nAChR activation on macrophages, suppressing NF-κB and elevating IL-10 (24–26). Within the present framework, this convergence is described as the IBR phase, where energy restoration aligns with immunological tolerance. The mitochondria-immune axis realignment during IBR is depicted in **Figure 2**.

The Mitochondria–Immune Axis during Informational Bio-Recalibration (IBR). Left: Inflammatory mitochondrial stress driven by TLR4*/*MyD88/NF-κB signaling with ROS elevation. Right: Nuclear-receptor-driven restoration (PGC-1α–TFAM–PPAR) producing IL-10 dominance, leading to systemic inflammation resolution, and restoration of redox homeostasis.

## 10 Conceptual interpretation of bio-intelligence in chronic inflammation

### 10.1 Mitochondria as informational processors

In this framework, mitochondria are conceptualized as bio-informational processors that decode metabolic and inflammatory inputs into adaptive electrical signals. Disruption of this signaling represents informational noise; restoration through IBR represents signal clarity.

### 10.2 Neural feedback and vagal synchrony

**Figure 3:**
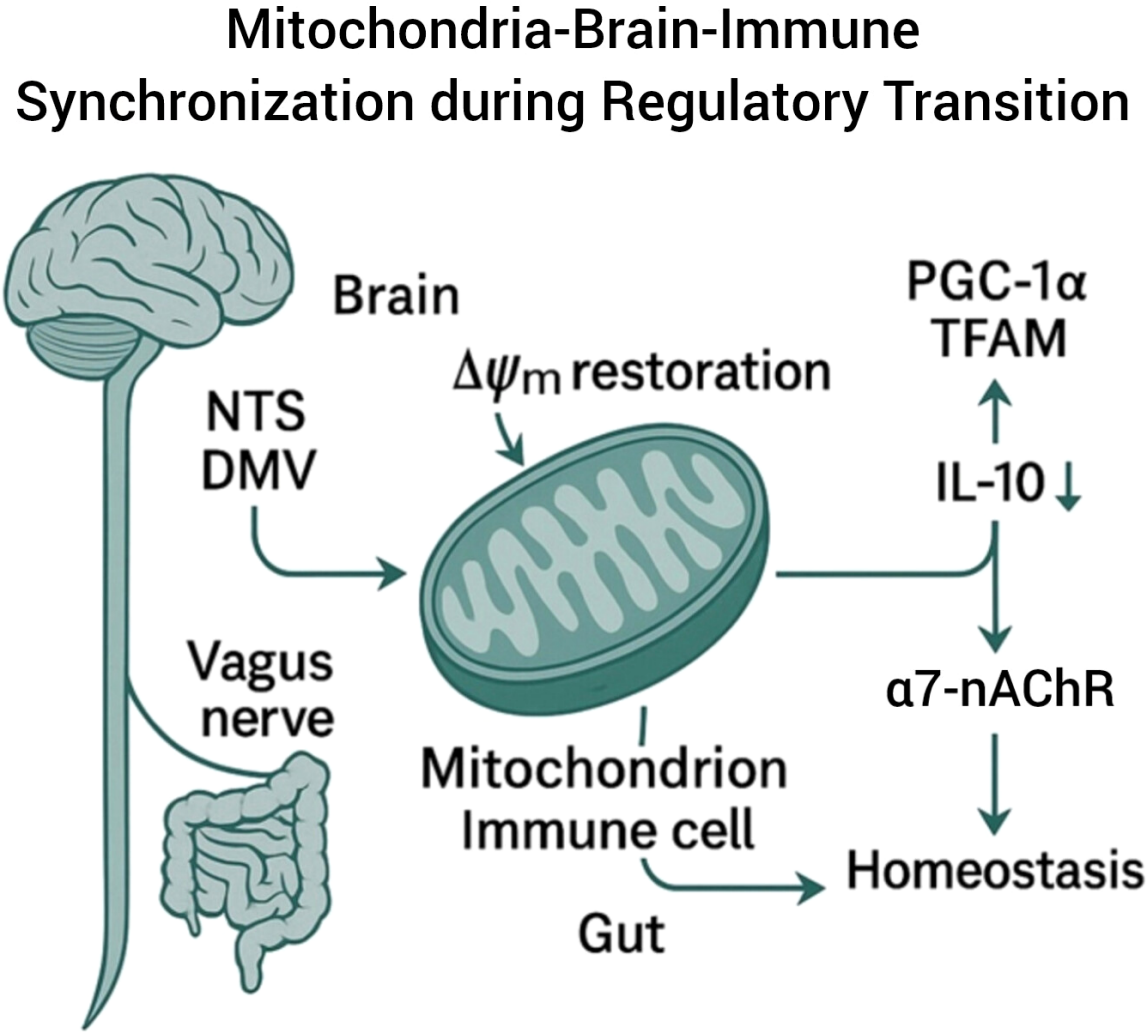
Mitochondria-Brain-Immune Synchronization via the Vagus Nerve during IBR.

The vagus nerve acts as a bidirectional conduit linking peripheral immune status with central autonomic nuclei — Nucleus Tractus Solitarius (NTS) and Dorsal Motor Nucleus of the Vagus (DMV) (27–30). Here, IBR is interpreted as occurring at the interface where afferent and efferent vagal feedback closes the mitochondrial–neural loop, transforming inflammatory signaling into coherent neural tone (28). This mitochondria-brain-immune synchronization model is illustrated in **Figure 3**.

The schematic illustrates the IBR framework highlighting vagus-mediated signaling to mitochondria within immune cells. Restoration of mitochondrial membrane potential (Δψm) and activation of nuclear transcription factors PGC-1α and TFAM enhance IL-10 release via the α7-nAChR pathway, promoting systemic homeostasis. The diagram emphasizes the neuro-immune coordination between the nucleus tractus solitarius (NTS), dorsal motor nucleus of the vagus (DMV), and peripheral immune circuits during mitochondrial recalibration. While the schematic highlights vagal cholinergic signaling as a primary illustrative pathway, complementary sympathetic efferent mechanisms (e.g., splenic β-adrenergic signaling) may also contribute to systemic neuro-immune regulation and are conceptually compatible with the proposed circuit.

### 10.3 Nuclear–mitochondrial coupling and energy re-entraining

Within this proposed regulatory circuit, nuclear receptors (VDR/PPAR) and mitochondrial transcriptional factors form a dual feedback system that modulates energy metabolism and immune response. PGC-1α acts as the bridge for information transfer between nuclear DNA and mitochondrial mtDNA (31, 32). IBR occurs when these axes resynchronize, transforming random oxidative bursts into structured electron flow that supports ATP synthesis and anti-inflammatory balance.

## 11 Discussion

### 11.1 The informational dimension of immunometabolism

Traditional immunology has focused primarily on cytokines and signaling molecules; however, the informational coherence that governs their orchestration remains underexplored. Within this framework, IBR is proposed as a conceptual bridge through which mitochondrial bioenergetics and neural regulation may transform molecular noise into structured, recoverable communication (27).

Rather than viewing the cell solely as a biochemical reactor, this perspective frames it as a semi-autonomous information processor in which energetic status, transcriptional responsiveness, and immune discrimination dynamically interact. In this context, chronic inflammatory persistence may reflect impaired signal discrimination and regulatory imbalance, rather than isolated hyperactivation of specific immune pathways.

By integrating mitochondrial redox dynamics with nuclear receptor coordination and neuro-immune feedback, the proposed framework expands contemporary immunometabolic paradigms toward a systems-level interpretation of inflammatory regulation.

### 11.2 Neuro-immune–mitochondrial convergence

Restoring coherence between mitochondrial and nuclear signaling may reduce oxidative stress while re-establishing immune tolerance. Within the proposed BIC framework, this transition is interpreted not as pharmacological suppression, but as systemic recalibration involving redox balance, transcriptional coordination, and vagal regulatory tone.

The vagal anti-inflammatory reflex is recognized as a neuro-immune regulatory pathway (28–30). The present framework extends this mechanism by positioning mitochondria as the functional endpoint where vagal signaling manifests physiologically. Activation of the α7-nAChR pathway and subsequent IL-10 signaling may reduce NF-κB activity and mitochondrial ROS production, contributing to closure of a homeostatic regulatory loop.

Elevated mitochondrial ROS is known to amplify NF-κB activation through redox-sensitive kinase signaling pathways (27, 33), thereby lowering inflammatory activation thresholds. Conversely, restoration of mitochondrial membrane potential (ΔΨm) has been associated with transcriptional reprogramming toward oxidative phosphorylation–dominant phenotypes and reduced pro-inflammatory gene expression (34, 35). These interactions provide a mechanistic bridge linking vagal signaling to mitochondrial-nuclear coordination.

This convergence of mitochondrial bioenergetics, nuclear transcriptional regulation, and autonomic feedback may provide a conceptual basis for understanding spontaneous remission phenomena reported in certain immune-related conditions. Importantly, this interpretation is presented as hypothesis-generating rather than deterministic.

### 11.3 Conceptual scope of the bio-intelligence circuit

The BIC is intended as a decision-organizing and interpretive framework for understanding chronic inflammatory dysregulation across biological systems. It does not prescribe fixed intervention sequences nor mandate specific therapeutic modalities.

Instead, BIC assists in identifying dominant regulatory failure domains by examining the interaction between mitochondrial energetic state, innate immune signaling tone, nuclear receptor transcriptional coordination, and autonomic regulation.

A core principle of this framework is the distinction between adaptive regulatory modulation and pathway-specific suppression. Rather than indiscriminately dampening immune activity, BIC emphasizes restoration of physiological coordination across regulatory systems. Inflammatory persistence is thus framed as a consequence of impaired signal discrimination and regulatory imbalance, allowing preservation of protective immune responses while reducing maladaptive inflammatory noise.

Evaluation within the BIC framework is inherently pattern-based, emphasizing convergence across multiple biological indicators rather than isolated quantitative thresholds.

Such systems-level regulatory restoration may be particularly relevant in the context of chronic inflammatory diseases, autoimmune disorders, and metabolic syndromes (33–35).

### 11.4 Limitations, boundary conditions, and falsifiability

The BIC framework is proposed as a systems-level regulatory model applicable primarily to chronic inflammatory dysregulation characterized by reversible network imbalance. It may not apply under conditions where structural or genetic constraints dominate biological behavior.

Boundary conditions where the BIC model would be expected to have limited applicability include:

- Acute septic shock or fulminant cytokine storm, where overwhelming systemic inflammation progresses independently of regulatory coherence restoration and requires immediate life-saving intervention.
- Primary mitochondrial DNA disorders or inherited oxidative phosphorylation defects, where fixed genetic impairments limit the capacity for bioenergetic recalibration.
- Advanced irreversible fibrotic or structural collapse, such as end-stage cirrhosis, pulmonary fibrosis, or extensive tissue scarring, where architectural destruction prevents restoration through regulatory modulation alone.

These boundary conditions define scenarios in which regulatory convergence may be insufficient to restore physiological homeostasis.

The framework may not apply under conditions of acute systemic collapse or irreversible structural damage. For example, in acute septic shock, primary mitochondrial DNA disorders, or end-stage fibrotic organ destruction, regulatory convergence may be insufficient to restore homeostasis.

#### 11.4.1 Explicit falsifiability clause

The BIC framework is explicitly structured as a falsifiable systems hypothesis. A prospective experimental design capable of disproving the framework would involve:

- Longitudinal measurement of mitochondrial bioenergetic markers (ΔΨm, ATP production, ROS dynamics),
- Nuclear transcriptional regulators (PGC-1α, TFAM, NF-κB target gene expression),
- Vagal/autonomic indices (heart rate variability, α7-nAChR signaling proxies),

in subjects undergoing a defined regulatory perturbation or intervention.

Failure to observe coordinated directional change across these domains despite controlled modulation would constitute empirical refutation of the convergence principle.

If sustained mitochondrial stabilization occurs without corresponding transcriptional reprogramming or autonomic coherence, or if inflammatory persistence continues despite demonstrable multi-domain regulatory restoration, the proposed convergence principle would be weakened or invalidated.

Similarly, persistent domain uncoupling under controlled perturbation would challenge the core assumption of systemic regulatory coupling.

Thus, the model generates testable predictions and is subject to empirical refutation under controlled experimental conditions.

The BIC is therefore presented as a testable systems hypothesis subject to empirical refutation under defined biological conditions.

### 11.5 Integrative outlook

This framework supports future expansion of IBR-oriented research through interdisciplinary and international collaboration. By integrating mitochondrial bioenergetics, neural feedback mechanisms, and immune recalibration concepts, the model offers a systems-level structure for studying multi-organ regulatory dynamics.

The integrated IBR map illustrates coordination between mitochondrial stability, nuclear transcriptional regulation, and vagal neuro-immune feedback, conceptualizing systemic recalibration as regulatory convergence across biological domains.

Such integrative modeling may help bridge observational clinical environments and translational research settings, enabling exploration of measurable remission pathways across chronic inflammatory phenotypes.

**Figure 4:**
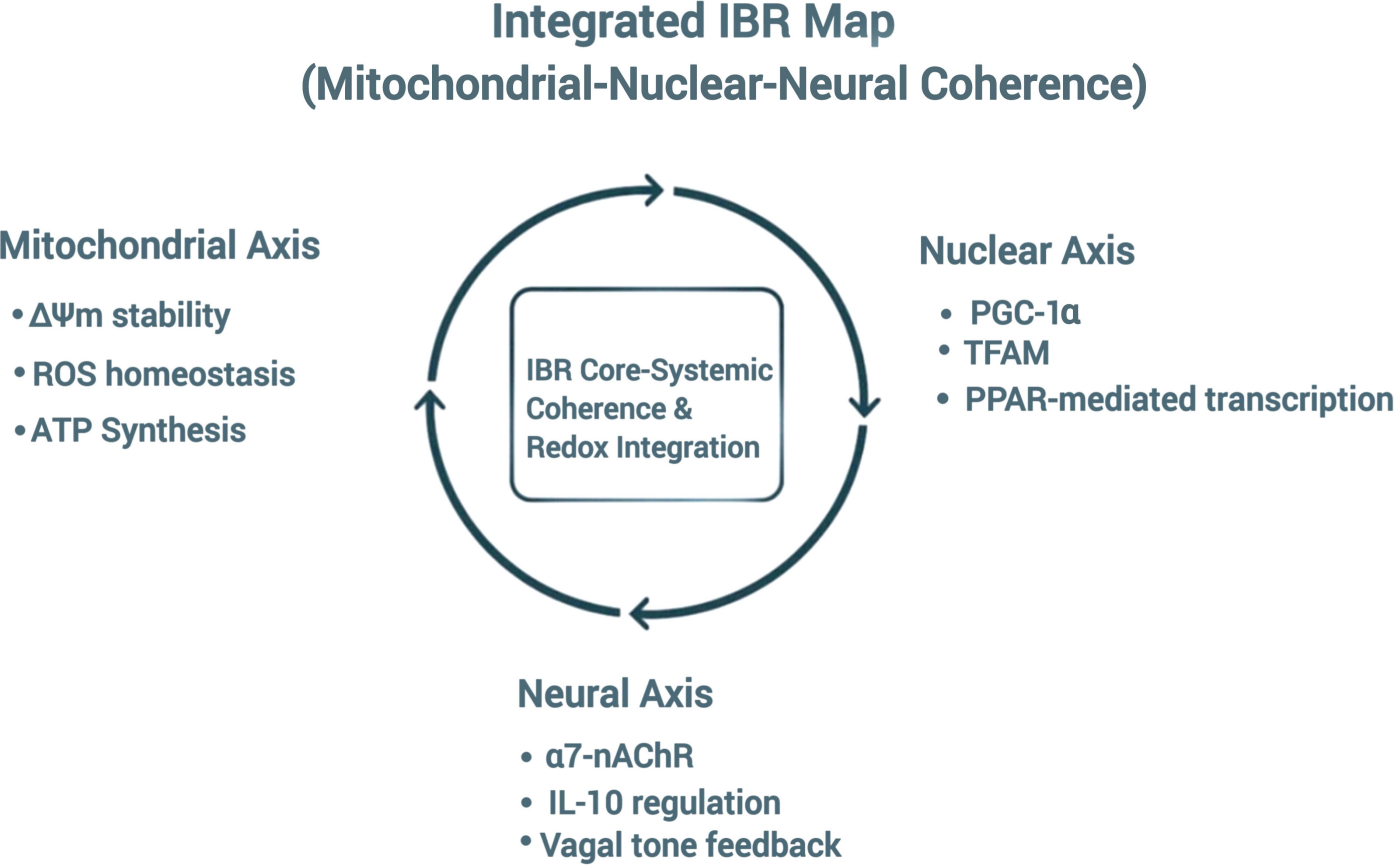
Integrated IBR map: Coordination of mitochondrial, nuclear, and neural coherence.

The progressive transition from mitochondrial degeneration (**Figure 1**) to systemic regulatory convergence (**Figure 4**) illustrates the proposed Informational Bio-Recalibration (IBR) continuum, linking intracellular bioenergetic recalibration to multi-organ homeostatic coordination.

Integrated IBR coordination Map illustrating feedback between mitochondria Δψm stability, oxidative homeostasis, ATP synthesis, nuclear receptor signaling (PGC-1α, TFAM, PPAR), and neural α7-nAChR, IL-10 regulation, vagal tone regulation. Informational loops converge to maintain systemic redox balance and immune homeostasis.

The schematic represents the BIC framework depicting the sequential restoration process from cellular signaling to systemic homeostasis. The cycle begins with intracellular signaling that initiates mitochondrial activation, progresses through mitochondrial recovery reflecting bioenergetic recalibration, and advances to neuro-immune synchronization denoting coordinated signaling between neuronal and immune pathways. The process culminates in a multi-organ regulatory loop symbolizing restoration of physiological stability within the IBR model.

### 11.6 Integrated bio-intelligence circuit model

**Figure 5:**
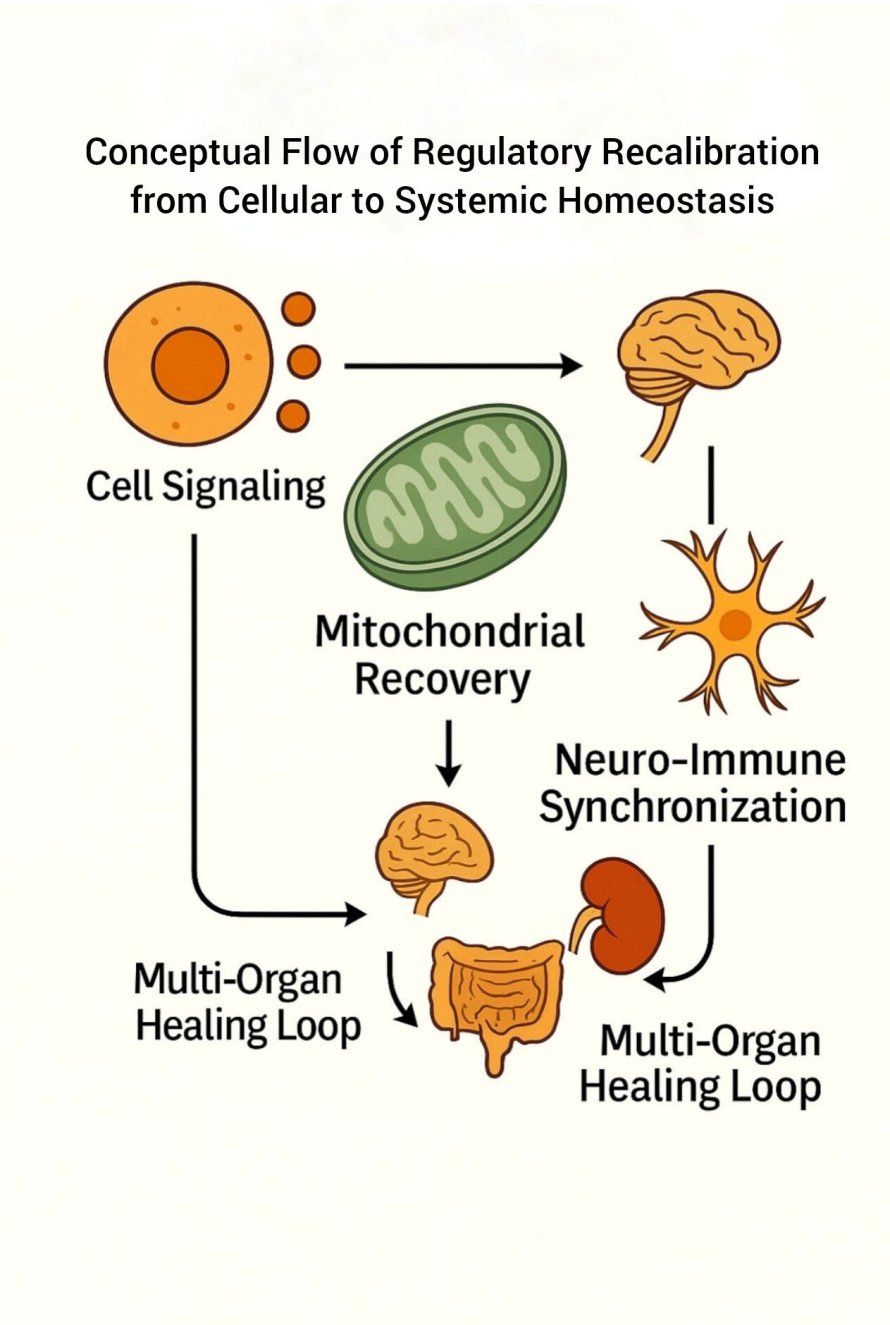
Flow of bio-intelligence from cellular recalibration to systemic homeostasis.

The overall mechanistic continuum of the Bio-Intelligence Circuit is summarized through the integration of **Figures 1**, **5**.

While **Figure 1** conceptually outlines the IBR model — illustrating the coordinated transition from mitochondrial degeneration to regeneration through mitochondria–nuclear–vagal signaling — **Figure 5** expands this concept into a systemic perspective, depicting the sequential flow of regulatory coherence from cellular signaling → mitochondrial recovery → neuro-immune synchronization → multi-organ regulatory stabilization.

This integration consolidates the IBR continuum, linking intracellular signal regulation with systemic immune coherence through an interconnected bio-intelligence network.

Together, these representations delineate a conceptual translational framework linking intracellular recalibration to whole-organism homeostatic coordination and provide a reproducible mechanistic structure for future investigation into immune regulation dynamics.

### 11.7 Clinical correlation signals supporting the framework

The conceptual development of the Bio-Intelligence Circuit (BIC) was informed by long-term exposure to diverse clinical environments, including orthopedic surgical settings, chronic inflammatory presentations, autoimmune-associated musculoskeletal conditions, and biomarker-guided outpatient evaluations. Across heterogeneous contexts, recurring physiological patterns were observed that aligned with the regulatory nodes described in the proposed model. For example, elevations in CRP, ESR, or IgE frequently corresponded with heightened inflammatory signaling; clusters of chronic fatigue and pain often coincided with markers consistent with mitochondrial stress and reduced autonomic tone; and hormonal irregularities commonly paralleled nuclear receptor and HPO-axis interactions.

These observations were retrospective, generalized, and entirely de-identified. They are not presented as clinical evidence but as experiential insights that contributed to refinement of the conceptual framework. The present work therefore offers a structured theoretical model intended to guide future systematic and experimental investigation of the proposed regulatory interactions.

## 12 Conclusion

The BIC presented in this manuscript offers an integrative, system-level hypothesis that links mitochondrial bioenergetics, LPS–TLR4–mediated innate immune activation, nuclear receptor dysregulation, and vagal inflammatory reflex collapse into a single, coherent model of chronic multi-organ inflammatory dysregulation. By conceptualizing inflammation as a disturbance in biological information processing rather than a purely immune-centric event, the proposed Informational IBR framework reframes chronic disease as a potentially reversible systems-level imbalance involving metabolic, neuroimmune, and transcriptional axes.

Importantly, the BIC framework is intended as a hypothesis-generating and interpretive scaffold rather than a prescriptive interventional or diagnostic model. It does not propose specific stimuli, treatment protocols, or quantitative thresholds, but instead delineates the regulatory conditions under which endogenous biological systems may regain coherent control. Within this framing, inflammatory persistence is understood as arising from impaired regulatory coordination rather than excessive immune function per se, allowing improvement without suppression of physiological immune defense.

While the present work does not derive therapeutic claims, the consistent mechanistic alignment observed across retrospective, de-identified clinical patterns supports the translational plausibility of this framework. The BIC therefore serves as a conceptual platform to guide future experimental design, observational validation, and systems-level investigation into multi-organ inflammatory dysregulation and recovery dynamics.

## Data Availability

The raw data supporting the conclusions of this article will be made available by the authors, without undue reservation.
